# Glycoproteomic and glycomic databases

**DOI:** 10.1186/1559-0275-11-15

**Published:** 2014-04-13

**Authors:** Deniz Baycin Hizal, Daniel Wolozny, Joseph Colao, Elena Jacobson, Yuan Tian, Sharon S Krag, Michael J Betenbaugh, Hui Zhang

**Affiliations:** 1 Department of Chemical and Biomolecular Engineering, Johns Hopkins University, Baltimore, MD, USA; 2 Department of Pathology, Johns Hopkins University, Baltimore, MD, USA; 3 Department of Biochemistry and Molecular Biology, Johns Hopkins Bloomberg School of Public Health, Baltimore, MD, USA

## Abstract

Protein glycosylation serves critical roles in the cellular and biological processes of many organisms. Aberrant glycosylation has been associated with many illnesses such as hereditary and chronic diseases like cancer, cardiovascular diseases, neurological disorders, and immunological disorders. Emerging mass spectrometry (MS) technologies that enable the high-throughput identification of glycoproteins and glycans have accelerated the analysis and made possible the creation of dynamic and expanding databases. Although glycosylation-related databases have been established by many laboratories and institutions, they are not yet widely known in the community. Our study reviews 15 different publicly available databases and identifies their key elements so that users can identify the most applicable platform for their analytical needs. These databases include biological information on the experimentally identified glycans and glycopeptides from various cells and organisms such as human, rat, mouse, fly and zebrafish. The features of these databases - 7 for glycoproteomic data, 6 for glycomic data, and 2 for glycan binding proteins are summarized including the enrichment techniques that are used for glycoproteome and glycan identification. Furthermore databases such as Unipep, GlycoFly, GlycoFish recently established by our group are introduced. The unique features of each database, such as the analytical methods used and bioinformatical tools available are summarized. This information will be a valuable resource for the glycobiology community as it presents the analytical methods and glycosylation related databases together in one compendium. It will also represent a step towards the desired long term goal of integrating the different databases of glycosylation in order to characterize and categorize glycoproteins and glycans better for biomedical research.

## Introduction

Glycosylation is a critical protein modification relevant to numerous physiological functions and cellular pathways. It is important for protein folding, signaling and stability in the circulatory system [1,2]. Alterations in the glycosylation site occupancy or glycan structures of glycoproteins have been associated with hereditary and chronic diseases such as cancer, diabetes, cardiovascular, inflammatory, neurological and neuromuscular diseases [3-5]. Indeed, the fields of glycopathology and glycophysiology are providing a broader understanding of disease genesis and progression [6]. Furthermore, glycoproteins have been extensively studied for the discovery of disease associated modifications that can be used for both diagnosis and/or therapy for these diseases [4,7].

Glycomics and glycoproteomics are two approaches used for the characterization of a specific cell, tissue or organ’s glycoproteome and glycome from an extracted protein mixture in a specific state. The glycoproteome is the full composition of glycoproteins in a specific cell or tissue type, while the glycome is the full set of protein-bound sugar groups. Glycomics focuses on the study of glycan structure whereas glycoproteomics focuses on glycosylated proteins and glycosylation sites. In glycoproteomic analysis, glycosylated proteins are first enriched with proper analytical techniques and then analyzed by LC/MS/MS for protein and glycosylation site identification. In glycomic analysis, the glycan moiety is often released from the glycoprotein and analyzed by mass spectrometry separately or in combination with chromatographic techniques. The chromatographic techniques can provide additional glycan identification and as well as the retention time of each identification. In addition, glycopeptides containing glycosylation sites and attached glycans can be analyzed by mass spectrometry without the release of glycans, which allows the identification of the glycosylation site and the specific glycans attached to the glycosylation site [8]. Initial works [9,10] and recent reviews have extensively discussed analytical techniques used for identification and quantification of both the glycome and glycoproteome [4,11-15]. Programs have recently being initiated both to merge current methodologies for identification of glycans or glycoproteome from complex tissues or cells and to establish databases for the identified glycosylated proteins [16,17]. Although many of the publicly available databases are dynamic and updated, they are not being used effectively because of a lack of common resources, websites, and public awareness. Collating all of these databases is critically important to the glycobiology community since data analysis is another key element in addition to analytical methods. This review summarizes the conventional methodologies used in glycoproteomic and glycomic studies and also assembles 15 different glycosylation related databases for the scientific community. Furthermore, this manuscript also introduces three glycoproteomic databases developed by our group: UniPep [18], GlycoFly [19] and GlycoFish [20].

## Glycoproteomic databases

Glycoproteomics is an emerging field which provides qualitative and quantitative information on a large number of glycoproteins. Recent improvements in glycoprotein isolation methods, bioinformatics, and mass spectrometry techniques have stimulated the subfield of proteomics known as glycoproteomic research [21].

In order to identify glycoproteins in a biological sample, the glycosylated proteins are first enriched with analytical, affinity, or chemical techniques. Subsequently, the type of glycosylation is determined. There are two major classes of glycosylation *N*-glycosylation and O–glycosylation. With N-glycosylation, the glycan group is attached to usually N4 residues of asparagines, whereas in *O*-glycosylation, the glycan group attaches to the hydroxyl oxygen of serine or threonine residues of a glycoprotein.

Emerging mass spectrometry techniques have significantly improved glycoproteomic studies. After the glycopeptides are enriched with a specific method, they can be qualitatively or quantitatively analyzed by tandem mass spectrometry to identify a large set of glycoproteins. A variety of technologies such as hydrazide chemistry, lectin chromatography or bead-immobilized techniques have been used for comprehensive analysis of site-specific glycosylation [22-26]. Although there are organized and structured databases for the proteomes and genomes of organisms which are complementary to each other, there is an absence of a unified, structured database for glycoproteome and glycome of organisms. Fortunately, a number of groups have established dynamic, publicly available databases to share their glycoprotome data [18,27,28]. Below are two tables, Tables 1 and 2, listing many of the databases concerned primarily with glycoproteomics and glycomics.

**Table 1:** Summary of glycoproteomic databases

**Database**	**Type**	**Species**	**Method**	**Entries**
**Unipep**	*N*-Glycosylated proteins and peptides	*Homo sapiens*	Hydrazide chemistry & Solid Phase Extraction, in-silico triptic digestion of IPI proteins, and prediction of NXS/T glycosylation site with proteotypic potential	2265
**Glycofly**	*N*-Glycosylated proteins and peptides	*Drosophila melanogaster*	Hydrazide chemistry and Solid Phase Extraction	740
**Glycofish**	*N*-Glycosylated proteins and peptides	*Danio Rerio*	Hydrazide chemistry and Solid Phase Extraction	269
**GlycoSuiteDB**	O-linked and N-linked Glycoproteins and glycans		Published glycoproteins with different methods	9436
**GlycoProtDB**	N2 and mouse tissues *N*-Glycoproteins	*Caenorhabditis elegans*	Lectin Concavilin A Chromatography	1465
		*Mus Musculus*		
***O*-GlycBase NetOGlyc**	*O* and C-Glycosylated proteins	Combination of references	Data curation from literature and coupling ZFN gene targeting, SimpleCell and Lectin Chromatography	2413
				3000
**dbOGAP**	*O*-GlcNAcylated proteins	*Homo Sapiens*	Curation from literature and SVM based prediction	798 (exp) 300 (pred)
		*Mus Musculus*		
		*Rattus Norvegicus*		
		*Drosophila Melanogaster*		
		*Xenopus Laevis*		

**Table 2:** Summary of glycomic databases

**Database**	**Type**	**Method**
**(CFG) Glycan Structure DB**	Glycan and glycan binding proteins	Glycan array screening, Glycan profiling
**GlycoBase**	*N* and *O*-linked glycan structures	HPLC based and MS based glycan analysis
**GlycomeDB**	Carbohydrate structures	Carbohyrate data from CFG , KEGG, BCSDB, Carbbank
**GlycoGeneDB**	GlycoGenes and mRNA expression	In-silico collection of cloned and characterized human glycogenes
**Glycan Mass Spectral DB**	*N*-and *O*-linked glycans, and glycolipid glycans	Glycan glycosidase digestion and analysis by HPLC with fluorescence or MS^n^ analysis
**Lectin Frontier Database**	Glycan-lectin interactions	Frontal affinity chromatography with fluorescence detection

### UniPep

The detection and interpretation of the changes in organ and plasma proteomes may provide information and insights for delineating disease states. For this reason, it is important to discover serum or organ-specific biomarkers for early detection of the disease. Profiling the glycoproteome of plasma and organs is promising because changes in the pathological or physiological state of the human body can be manifested by aberrant glycosylation [18,24]. Zhang et al. conducted a study to connect the organ and plasma proteomes using the hydrazide chemistry method to capture the *N*-glycosylated proteins [24] of plasma, bladder, breast cancer cells, liver, lymphocytes, cerebrospinal fluid, prostate tissue and prostate cancer cells [18]. In this study, 2265 unique *N*-linked glycosylation sites were identified with high confidence and these glycosylation sites and associated glycoproteins are publicly available within the UniPep website (http://www.unipep.org) [27]. In addition, thousands of unique *N*-linked glycosites from different mouse tissues were also reported [29-31]. The database for mouse *N*-linked glycosites can be developed using a similar process. Thus, UniPep provides access to human and mouse *N*-glycosylated proteins and their *N*-glycosylation sites for biomarker discovery. All the proteins including their protein ID are listed on this dynamic website. Furthermore, the website provides information on all these *N*-glycosylated proteins including identified *N*-glycosylated peptide sequences and probability scores.

Moreover, the consensus *N*-glycosylation sites of the proteins can be reached from this database. The database provides the in silico trysin digest of the proteins and the possible NXS/T motifs. Another bioinformatics tool in this website determines whether these glycosylation sites can be detected or not in an MS/MS experiment which is an important guide for the experimental design. As a next phase of the project, this library of theoretical peptides, which have already been scored for their likelihood of mass spec detection, will be compared to the experimentally deposited proteotypic peptides from a variety of LC/MS/MS experiments.

### GlycoSuiteDB

Unicarbkb (http://unicarbkb.org) provides information on both the glycan structure and glycosylated peptides of proteins [32,33]. This database includes all the published glycan types and glycosylation site information found throughout the literature from 1990 to 2005. Currently, there are 9436 entries from 864 references belonging to 245 species, including *Homo sapiens, Rattus norvegicus and Mus musculus.* On the website, proteins of interest can be searched by name, Uniprot, SwissProt or TrEMBL accession numbers. The database provides access to information such as the biological source of the protein, its glycosylation sites and possible glycan structures at those sites for both *N*-glycosylated and *O*-glycosylated proteins. Furthermore, it includes literature references and the relevant links to PubMed. The methods used for the identification of the glycans and glycosites are also provided on the website. Finally, glycoproteins associated with particular disease states in the literature are provided [34,35]. While a major disadvantage of GlycoSuiteDB is that it has not been updated since 2005, it was recently incorporated as part of UniCarbKB [33]. Since UniPep and GlycoSuiteDB are excellent sources for biomarker and therapeutics discovery, methods should be implemented to update and provide glycoproteomes of more organisms in addition to those currently catalogued.

### GlycoFly

**Figure 1:**
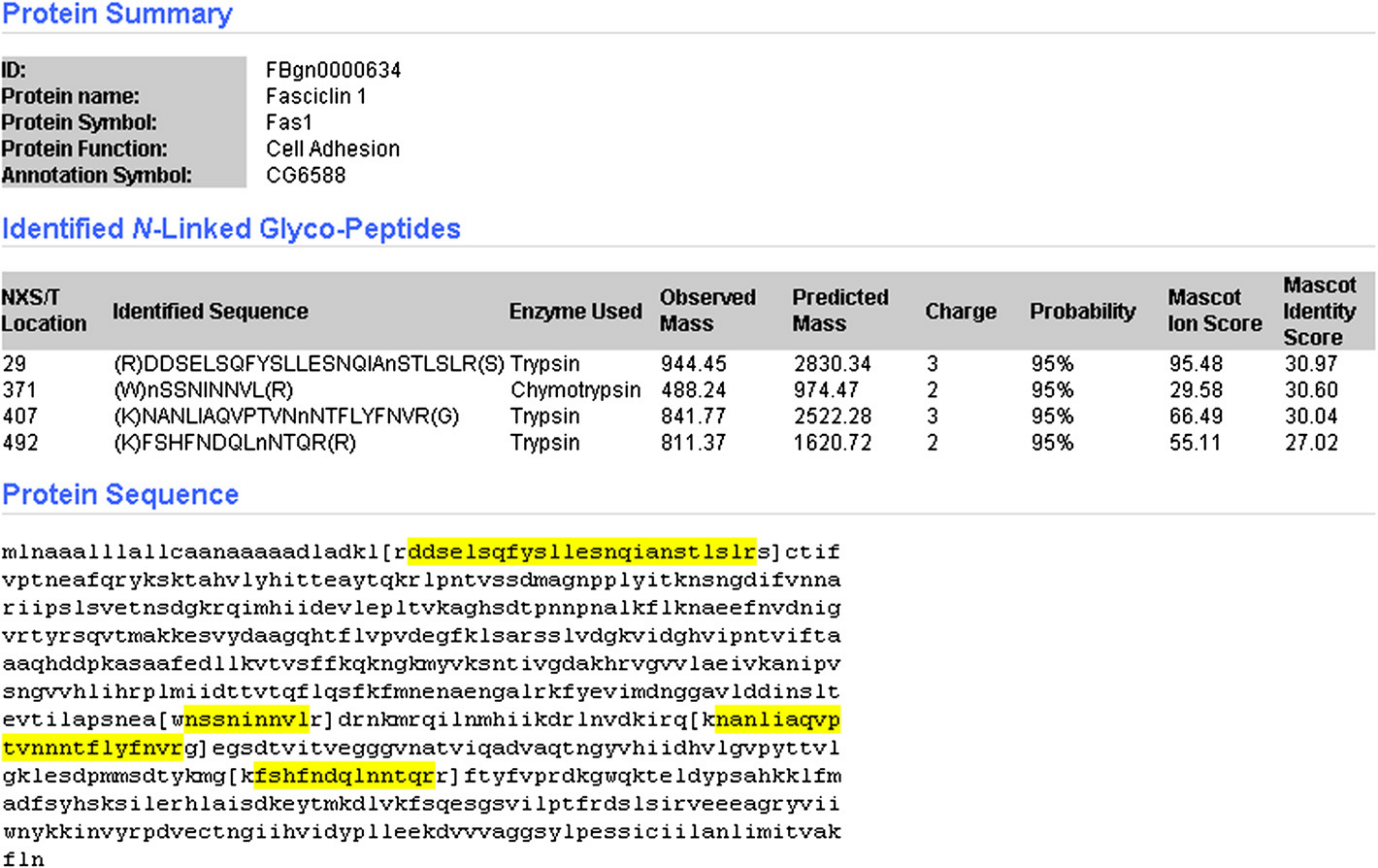
**Example of GlycoFly website protein, Fascilin 1 (**http://betenbaugh.org/GlycoFly/**).**

GlycoFly is another publicly available database for *N-*glycosylated proteins and peptides of *Drosophila melanogaster *[19]. *Drosophila* is an important model organism to study since it is often applied to interpret the effects of gene mutations on human diseases. For instance, a mutation in the volado/scab glycoprotein gene, which leads to glycan variations, has been shown to cause memory deficits [36] and a mutation of the wolknauel gene of the glycosylation pathway has resulted in disruptions in embryonic patterning [37]. Furthermore, blood nerve barrier dysfunction and loss of glial septate junctions in the peripheral nervous system have been observed when contactin, neuroglian, and neuroxin IV genes are mutated [38]. These proteins are highly glycosylated and localized to the nervous system of flies [19]. As a result, GlycoFly has focused on glycoproteome identification of the central nervous system of flies. Four hundred and seventy seven central nervous system glycoproteins containing 740 NXS/T glycosylation sites were identified. This information is available publicly on the GlycoFly website (http://betenbaugh.org/GlycoFly/) [39]. The proteins are listed with their Flybase IDs, and a specific protein of interest can be searched by name or sequence. The function of each protein, identified glycosylated peptide sequence and its probability are compiled as well. An example output from the website is displayed in Figure 1. The relative publications and an overview of the experiments as well as in-silico prediction tools and links to other glycoproteome databases are not yet active in this database.

### GlycoFish

*Danio rerio* (zebrafish) is a promising model system to understand vertebrate development and human disease because of biological and functional similarities between humans and zebrafish. Larval and embryonic zebrafish have also been used to explore potential therapeutics for developmental disorders since some pharmacological agents, especially neurotoxins and neuroprotectants, have shown similar effects in zebrafish and humans [40,41]. Furthermore, mutations in zebrafish cause diseases that resemble human diseases; for example, both adult and embryo zebrafish have been used to understand neurological and neuromuscular diseases such as Huntington’s, Alzheimer’s and Parkinson’s. [42-44]. Therefore, the glycoproteome of zebrafish embryos was characterized by our group in order to determine *N*-glycosylated sites of proteins present during in vertebrate development [20].

Using the hydrazide chemistry method, 169 *N*-glycosylated proteins were identified. These proteins include 269 *N*-glycosylation sites found on 265 *N*-glycopeptides. In order to make this data publicly available, the GlycoFish database (http://betenbaugh.org/GlycoFish/), which [45] lists the mass spectrometer properties of identified *N*-glycopeptides and gives functional and sequential information on the identified *N*-glycosylated proteins, has been established. This database can be further improved by in-silico prediction of glycosylation sites as well as addition of related publications, overview of the experiment, and links to the other glycoproteomic databases.

### GlycoProtDB (GPDB)

GlycoProtDB (http://jcggdb.jp/rcmg/gpdb/index.action) is a database for the *N*-glycoproteins of *Caenorhabditis elegans* N2 and mouse tissues, identified from lectin chromatography experiments [46,47]. In order to enrich the *N*-glycosylated proteins, lectin affinity column based isotope coded glycosylation site specific tagging (IGOT) was used. The proteins were digested, applied to a lectin affinity column in order to enrich the *N*-glycosylated proteins, and *N*-glycanase treatment was performed to remove the glycosylated peptides in ^18^O-labeled water for tagging of the asparagine sites converted aspartate sites [48-50]. Then shotgun analysis with LC/MS/MS identified 400 N-glycosites on 250 glycoproteins using this elegant technique in the initial study [48]. These numbers were increased to 1465 *N*-glycosylated sites on 829 proteins in subsequent studies [50]. Furthermore, 1200 mouse liver glycoproteins, accessible in the GlycoProtDB database [49] were also identified using 2D-LC-MS/MS studies.

Proteins of interest can be searched on GlycoProtDB by their name, amino acid length, molecular weight or database identifiers. A user friendly website provides information on the glycoprotein ID, amino acid sequence, and experimentally identified glycosylation sites of the proteins. It also provides access to the method and lectins used for the identification of these glycopeptides [46,47].

### *O*-GlycBase

*O*-GlycBase (http://www.cbs.dtu.dk/databases/OGLYCBASE/) is a prediction website of the Technical University of Denmark (DTU) [51,52]. This database includes 242 proteins with 2413 *O*-glycosylation sites and relevant references. *O*-glycosylated proteins were documented to establish a network for predicting the *O*-GalNac sites of the proteins [53]. This prediction database for the mucin-type *O*-glycosylated proteins is named NetOGlyc (http://www.cbs.dtu.dk/services/NetOGlyc/) [54], which identifies potential *O*-glycosylation sites for any submitted protein with 76% confidence [53,54]. Furthermore recently NetOGlyc4.0 model has been developed which is based on the first *O*-glycoproteome map of human consisting of 3000 *O*-glycosites from over 600 *O*-glycoproteins using genetic engineering approach [55-57]. O-Unique (http://www.cbs.dtu.dk/ftp/Oglyc/O-Unique.seq), another database established by DTU, includes 53 mucin type mammalian glycoproteins with 265 experimentally proven *O*-glycosylation sites [58].

### dbOGAP

*O*-GlcNAcylation is the addition of β-N-acetylglucosamine (GlcNac) to Ser or Thr aminoacids by the *O*-GlcNac transferase (OGT) enzyme. Unlike mucin type *O*-glycosylation, GlcNAc attachment occurs only for nuclear and cytoplasmic proteins with no further addition or extension of carbohydrates. *O*-GlcNAcylation plays an important role in biological processes and has been associated with diseases such as diabetes, cancer, and neurodegeneration. For this reason, dbOGAP (http://cbsb.lombardi.georgetown.edu/OGAP.html) database for *O*-GlcNAcylated proteins and sites was established and a support vector machine (SVM) based sequence program to predict the protein *O*-GlcNAcylation sites was developed [59]. This database includes 798 experimentally proved and 365 predicted proteins of human, rat, mouse, frog and fly [60]. For each protein entry, the experimentally characterized or predicted *O*-GlcNacylation and phosphorylation sites are available at this website, along with the molecular and biological function of each protein and its importance in disease states. The *O*-GlcNAcScan feature allows users to predict *O*-GlcNacylation sites for any submitted protein [59,60].

### Glycomic databases

Both the glycosylation sites and the bound glycan structures represent important aspects of systems glycobiology. More than 200 glycosyltransferases are responsible for the addition and modification of carbohydrates with different linkages in order to generate a wide range of diverse glycans [61]. As a result, glycan characterization can be challenging due to the heterogeneity and complexity of oligosaccharide moieties. However, specific carbohydrates can play key roles in cell-cell recognition, receptor-ligand binding, protein interactions, and protein stability *in vivo *[62]. In recent years, high-throughput glycomic techniques have enabled fast and robust glycan characterization to demonstrate lot-to-lot consistency in pharmaceutical therapeutics and to understand the role of glycans in human disease [62].

Complete glycan profiling can include the detection, identification, and quantification of the carbohydrates as well as the the identification of linkages between specific monosaccharides. Different methods including chromatographic separation and mass spectrometry [63] are used for the analysis of glycans. Glycan analysis from a biological sample requires the release of an intact glycan from the protein followed by separation and detection using chromatography or mass spectrometry based glycan methods. Various combinations of methods are also used in glycan isolation and characterization as summarized in recent articles [62-72].

Evaluating glycans can represent a more complex task than proteomics or genomics because of the multiple glycosyltransfers that occur during glycan biosynthesis. Furthermore, various *O* and *N*-glycan structures are possible depending on the specific target proteins and glycosyltransferases present, making decoding the glycans challenging [63,73]. To enhance knowledge of glycomic patterns, glycomic databases are being established that document the different glycan structures and make this information publically available [73]. A table summarizing the various databases primarily concerned with glycomic studies is listed below.

### Consortium Functional Glycomics (CFG) glycan structure database

CFG provides one of the largest databases for understanding the roles of carbohydrates in cell communication [28]. It also includes a glycan structural database (http://www.functionalglycomics.org/glycomics/molecule/jsp/carbohydrate/carbMoleculeHome.jsp) in order to compile and integrate glycomic data sets for the glycoscience community [74]. CFG has provided both core facilities for data generation and a bioinformatics platform for annotating glycan structural data [75]. The analytical glycotechnology core facility of CFG has profiled permethylated *N*- and *O*- glycans for human and mouse tissues and cell lines. In addition, CarbBank and Glycominds, which include *N*- and *O*-glycans analyzed in other studies, are integrated in this database. Different options to search for glycans of interest include their name, composition, molecular weight, Glycan ID, IUPAC ID, the cell line or tissue sample. Both basic and complex searches can be performed depending on the bioinformatics goals. For example, one can search for glycans containing sialic acid or those associated with human cancer. When selecting the glycan of interest, the glycan cartoon and IUPAC 2D structures are shown and its properties, such as molecular weight, are listed. Furthermore, CFG identifies whether this glycan is *N*- or *O*-linked and studies related to this glycan are noted in the reference section [74,75]. The substructure search option is another uncommon and useful feature of CFG database. The substructure interface provides different common carbohydrate motifs, for *O*-linked and *N*-linked glycans that can be modified or extended to form the desired glycan structure [74,75].

### GlycoBase

Fluorophore labeling using 2-aminobenzamide (2-AB) is often used for labeling the glycans for subsequent HPLC analysis. A 2-AB labeled dextran ladder was used to assign glucose unit (GU) values based on the retention times of glycans [76]. GU values representing the HPLC retention times for more than 350 glycan structures are available on the GlycoBase database (http://glycobase.nibrt.ie/glycobase/show_nibrt.action) [77]. In addition to the GU values, monosaccharide compositions and their linkages are represented with pictures for each glycan. Each entry has links for the exoglycosidase digestion products and the groups where the glycan of interest can be found. Also, relevant publications related to these glycans are listed as references [76,78].

GlycoBase also includes the GlycoExtractor interface for extraction of HPLC glycan data into a common format [79]. GlycoExtractor can export the peak areas and GU values from large sets of HPLC data in order to integrate shared data in the same format. This format makes data analysis and storage easier for glycan profiling, which is helpful for biomarker discovery and generation of therapeutics [80].

### GlycomeDB

GlycomeDB is a database established for the integration of the carbohydrate structures and annotations from seven different publicly available databases (CFG, Bacterial Carbohydrate Structure Database (BCSDB), GLYCOSCIENCES.de, Kyoto Encyclopedia of Genes and Genomes (KEGG), EUROCarbDB and Carbbank) [81]. GlycomeDB also introduced both GlycoCT and GlycoUpdateDB interfaces. GlcyoCT is a universal data format established for the incorporation of glycan datasets onto the GlycomeDB website. GlycoUpdateDB interface generates updates from different databases to the website on a weekly basis. After downloading the datasets from public databases, GlycoUpdateDB translates the data into the GlycoCT format and integrates the new data into GlycomeDB website. More than 35,873 different carbohydrate sequences have been uploaded in GlycoCT format with 11,822 structures fully determined including all linkage positions, base type, anomers, ring size and modifications [82,83]. GlycomeDB provides the image of the glycan structure, its specifications in GlycoCT format, and links to the external databases for further information on the glycan of interest. It is also possible to learn all the identified oligosaccharide structures for a particular species. When searching a specific species, the website lists the glycans with their cartoon representations and references [81].

GlycomeDB has also absorbed another important database: the Japan Consortium Glycobiology and Glycotechnology Database (JCGGDB) (http://jcggdb.jp/index_en.html), which itself is composed of the GlycoGene Database (GGDB) (http://jcggdb.jp/rcmg/ggdb/) and Glycan Mass Spectral Database (GMDB) (http://jcggdb.jp/rcmg/glycodb/Ms_ResultSearch) [84-86]. The JCGGDB database provides a different approach for displaying glycomic information compared to other available databases.

GGDB includes all the identified genes related with a glycosylation pathway such as glycosyltransferases, sialyltransferases, carbohydrate transporters and synthases. All the DNA and mRNA sequences of these enzymes with their gene expression profiles in tissues are included as well. Furthermore, graphical representations of the substrate specificities are also provided [84]. The GMDB approach is similar to the GlycoBase approach for the identification of glycans. However, instead of GU values, GMDB provides spectral view of glycans obtained with MALDI-QIT-TOF MS. Each carbohydrate structure has an MS^n^ fragmentation pattern and these collision-induced dissociation spectra are stored in the database to enable spectral matching and glycan identification. The MS^n^ spectra of any glycan can then be searched based on its m/z value or composition. The website also provides an option to include modifications such as phosphorylation on the glycan of interest. If the glycan is coupled with a fluorescent reagent, such as 2-aminopyridine, this can also be included in the list of labeling groups to look for the specific spectra of 2-aminopyridine coupled glycans [85,86].

### GlycoSuiteDB

In addition to being a glycoproteome database, GlycoSuiteDB, established by Tyrian Diagnostics Ltd provides access tomore than 3238 unique carbohydrate structures from 245 different species. GlycoSuiteDB is a web-friendly database which provides information on the mass and composition of the glycan, the linkages and the anomeric configuration. This database gives detailed information on the cell line or tissue in which each glycan structure is found, as well as the method used to determine the specified glycan, its role in disease states or therapeutic production, and links to references [32,34,35].

The website also lists all the available glycan types in the database with a particular composition or mass. In addition, one can construct or extend a structure and then look up if that specific carbohydrate has been identified or investigated in the literature. Another search option available is the ability to find glycans associated with a specific biological source or disease. For example, when performing a search with blood as your biological source, 49 different glycans are specified [32].

### EuroCarbDB

EUROCarbDB (https://code.google.com/p/eurocarb/) is a European based core database for the collection of carbohydrate data and the development and housing of corresponding bioinformatics tools [87]. This initiative has been established to provide the technical infrastructure needed for standardization of the glycomic data and the appropriate analytical tools. EuroCarbDB aims to compile large, high quality primary research data sets from MS, NMR and HPLC experimental work into a single location in order to create common standards for storing these datasets. In conjunction, EuroCarbDB has established bioinformatic tools for analyzing, processing and identifying the glycan structures from MS, NMR spectra and HPLC profiles. For example, a software tool has been developed, GlycanBuilder, which can be used to visualize, display and assemble glycan structures with a symbolic notation. GlycanBuilder can either be used in a user-independent manner to display glycans or as a user-dependent tool to draw specific glycan structures [88]. In addition, GlycoWorkbench is another glycoinformatics tool which can be used to annotate the *N* and *O*-glycans from mass spectra data [89]. One of the challenges in glycomics databases has been the digital representation of carbohydrate structures in a computer readable format. Two glycobioinformatics tools, Glyco-CT and Glyde have been established for encoding the glycan structures. Recently Glyde has been recognized as the standard format for the exchange of information between databases [88]. Besides these, Glyde II and Glyde II DTD were developed by University of Georgia. Glyde II DTD especially provides the preservation of partonomy and granularity in the carbohydrates [90].

## Databases for Glycan-protein interactions

Glycan-Binding Proteins (GBP) such as antibodies, lectins, and receptors has been used for glycan recognition over many years. However, determination of specificities of GBPs required a large amount of the glycans and much labor-intensive preparation prior to the development of glycan microarray technology. Glycan microarray technology has since accelerated studies in glycomics since glycan binding specificities can be analyzed quantitatively in a short period of time using much smaller amounts of sample material [91].

The most widely used highthroughput method for glycan profiling are lectin microarrays, which can analyze multiple lectin-glycan interactions simultaneously [92-94]. Antibodies are also used in glycan microarrays since they can be specific to particular carbohydrate epitopes. Antigenic epitopes such as Lewis x and Sialyl Lewis A can be strongly recognized by specific monoclonal antibodies [73,91,95]. However, antibodies are usually unable to differentiate between *O*-glycans, *N*-glycans or glicolipids. They typically bind to their specific epitopes regardless of the glycan type [95]. The methods that are used in glycan microarrays and available databases are discussed below.

### CFG

The Consortium for Functional Glycomics (CFG) group also has a Protein-Carbohydrate Interaction Core facility which applies two different methodologies for protein analysis and glycan recognition. Both microwell based and glass slide arrays similar to DNA microarrays are used to screen hundreds of glycans, lectins, antibodies and pathogenic proteins. Streptavidin-coated wells are covered with biotinylated synthetic or biological glycans to identify novel carbohydrate binding ligands. Moreover, glycan printing on the *N*-hydroxysuccinimide-reacted glass slide arrays is being used to expand number of possible glycan ligand targets. This technology also has an advantageous low signal to noise ratio [74].

The CFG database allows users to search through plate, printed and pathogen arrays for the specific analyte of interest. Numerous animal lectins such as C-type lectins, siglecs, galectins as well as plant lectins, pathogens, microbial lectins, antibodies, serum, cells and organisms are available under the analyte category. When the analyte of interest and array type are chosen, the website finds all the studies related to them. The primary glycan binding specificity, ligand site and any information related to this glycan binding protein are also provided in the database [96,97].

### L*f*DB

**Figure 2:**
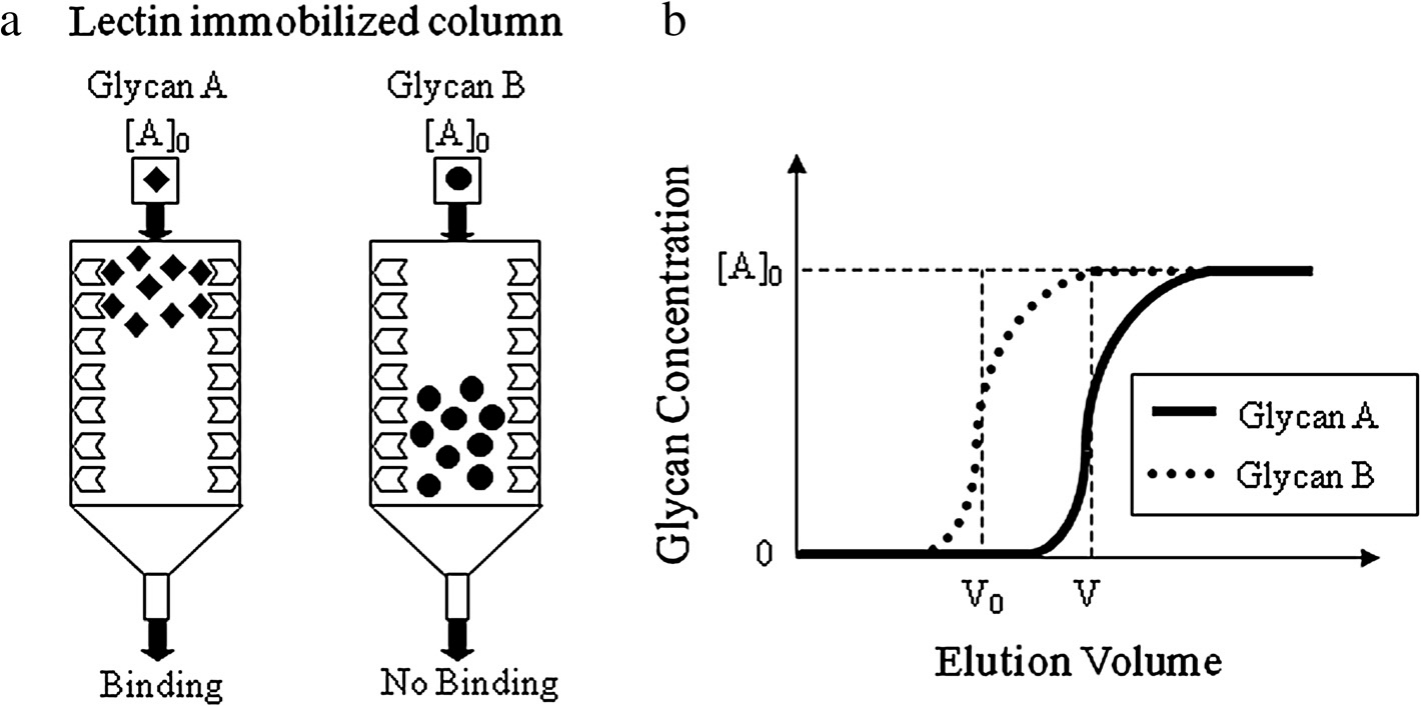
**Frontal affinity chromatography for the quantification of lectin-glycan constants.** Schematic graphs of **a)** lectin immobilized column **b)** isocratic elution system.

The Lectin *Frontier* Database (L*f*DB) was established by JCGGDB and provides quantitative information on glycan-protein interactions. The binding specificity of each lectin to different glycans is variable and this affinity can be quantified in terms of an association constant (K_a_). Frontal affinity chromatography with fluorescence detection (FAC-FD) is a common method used to determine affinity constants since it produces reliable and reproducible data [47]. As shown in Figure 2, Langmuir’s adsorption principle is applied in this isocratic elution system.

Pyridylaminated glycans (PA-glycans) in low concentrations can be loaded onto the lectin-immobilized column, and the binding specificity of a glycan calculated based on the change in the volumes as shown in the following equations.

$$K_{d}=\frac{B_{t}}{\left(V-V_{0}\right)}-\left[A_{0}\right]\;\mathrm{and}\;K_{a}=1/K_{d}$$

Where K_a_ is the affinity constant, B_t_ is the effective lectin content, [A_0_] is the initial glycan concentration, and V-V_0_ is the difference between the initial glycan volume of the glycan of interest and a negative control [92].

In LfDB (http://jcggdb.jp/rcmg/glycodb/LectinSearch), a variety of lectin affinities towards glycans are available. Any lectin type or monosaccharide specificity can be searched. Once the glycan binding protein is found, all the information related to this protein and its K_a_ values toward different glycans can be obtained from this database [98].

## Conclusion

Fifteen different glycomic and glycoproteomic related databases are described in the current study. These databases include more than 30,000 entries for experimentally identified or predicted glycans and glycopeptides. The structural information on the glycan or glycosite of these glycoproteins and hyperlinks to their references are also provided in these databases. Each of these databases has key features. For instance Unipep includes both experimentally proven glycoproteins and their glycosites and also in-silico predicted glycosites on human proteins. GlycoFly focuses on the N-glycosylated peptides of *Drosophila melanogaster* whereas GlycoFish provides the list of N-glycosites of zebrafish. O-GlycBase, dbOGAP are the specific databases for O-glycosylation and O-GlcNAcylation. CFG and EuroCarbDB are the two largest databases for carbohydrates whereas GlycoBase and GlcyomeDB databases include extensive information on the glycans. Furthermore databases such as CFG and LFDB provide information on the glycan-protein and lectin interactions. This review will be a useful resource for glycobiology studies and institutions searching for information on glycoproteins of interest. Furthermore, assembling the databases in this review and others will assist in the eventual formation of a single resource for glycomic and glycoproteomics high-throughput data. In the long term, the glycobiology community should strive to create a fully integrated and dynamic database that includes all the elements described in this review. One vision would be a database that has all the glycosylated proteins, indicating if they are O or N-glycosylated, and showing their *O* and *N*-glycosylation sites. We could then add additional functionalities to the database including all known glycan structures obtained at the designated glycosylation site together with specific glycosylation linkages. Of course, some of this data are not yet available, and thus there are additional experimental data and complementary bioinformatics that need to be obtained before a comprehensive glycomics database can become a reality.

## Competing interests

The authors declare that they have no competing interests.

## Authors’ contributions

DBH has done the literature search and drafted the review article. DW, JC and EJ have done extensive research for the collection of glycoproteomic databases and glycomic databases. They have worked on the figures and tables. YT has done research and has provided the drafting of the analytical methods used for glycoproteome identification. DW, SSK, MB and HZ edited and further improved the draft for publication. All authors read and approved the final manuscript.
